# Deep Learning-Based High-Frequency Ultrasound Skin Image Classification with Multicriteria Model Evaluation

**DOI:** 10.3390/s21175846

**Published:** 2021-08-30

**Authors:** Joanna Czajkowska, Pawel Badura, Szymon Korzekwa, Anna Płatkowska-Szczerek, Monika Słowińska

**Affiliations:** 1 Faculty of Biomedical Engineering, Silesian University of Technology, 41-800 Zabrze, Poland; pawel.badura@polsl.pl; 2 Department of Temporomandibular Disorders, Division of Prosthodontics, Poznan University of Medical Sciences, 60-512 Poznań, Poland; korzekwas@gmail.com; 3 Anclara sp. z o.o., 02-624 Warszawa, Poland; dr.platkowska@gmail.com; 4 Department of Dermatology, Military Institute of Medicine, 01-755 Warszawa, Poland; mslowinska@wim.mil.pl

**Keywords:** high-frequency ultrasound, inflammatory skin diseases, skin lesions, image classification, deep learning, transfer learning, Grad-CAM

## Abstract

This study presents the first application of convolutional neural networks to high-frequency ultrasound skin image classification. This type of imaging opens up new opportunities in dermatology, showing inflammatory diseases such as atopic dermatitis, psoriasis, or skin lesions. We collected a database of 631 images with healthy skin and different skin pathologies to train and assess all stages of the methodology. The proposed framework starts with the segmentation of the epidermal layer using a DeepLab v3+ model with a pre-trained Xception backbone. We employ transfer learning to train the segmentation model for two purposes: to extract the region of interest for classification and to prepare the skin layer map for classification confidence estimation. For classification, we train five models in different input data modes and data augmentation setups. We also introduce a classification confidence level to evaluate the deep model’s reliability. The measure combines our skin layer map with the heatmap produced by the Grad-CAM technique designed to indicate image regions used by the deep model to make a classification decision. Moreover, we propose a multicriteria model evaluation measure to select the optimal model in terms of classification accuracy, confidence, and test dataset size. The experiments described in the paper show that the DenseNet-201 model fed with the extracted region of interest produces the most reliable and accurate results.

## 1. Introduction

Ultrasound (US) is described in Reference [1] as a powerful and ubiquitous screening and diagnostic imaging technique, which has found applications in different fields. Being non-invasive, convenient and safe, it is widely used in multiple organ examinations, prenatal screening, or guided biopsies. Its fast development, resulting in high-frequency ultrasound (HFUS, between 20 and 30 MHz) and ultra high-frequency ultrasound (UHFUS, >30 MHz), has opened up new opportunities for medical applications [2]. It is gaining popularity in dermatology, dermatological oncology, ophthalmology, cosmetology, and aesthetic medicine. US at frequencies below 100 MHz is now commonly used in medical practice [3], and the >100 MHz probes are constantly being designed [4]. Improving spatial resolution of acquired images, the higher frequency of the US probe enables clear visualization of superficial structures such as the fat layer, the muscle layer, blood vessels, hair follicles, and skin appendages [2,5,6,7]. HFUS is used for healthy skin analysis, where skin thickness is inversely proportional to age, and due to increased collagen production connected with aging, echogenicity tends to increase too [2]. It can be used to estimate the hair follicle growth phase, identify their inflammation and early signs of adnexal pathologies [2]. HFUS is a reliable method with outstanding intrahand interreproducibility for measuring melanoma depth in vivo and may enable single-step surgical excision [8]. The use of ultrasound helps in therapeutic decisions and surgical planning for non-melanoma and melanoma skin cancers and detecting early neoplasms [2]. Kleinerman et al. [9] demonstrate its potential for differentiation between melanoma, benign nevi, and seborrheic keratoses, along with the monitoring of inflammatory conditions and photo damage.

One of the first HFUS applications in dermatology can be found in inflammatory skin diseases [2], where it has opened up new opportunities in diagnosis and treatment monitoring. Both HFUS and UHFUS enable reliable, accurate, and fast skin layer analysis. As reported in [2], the first implementation in inflammatory skin diseases was scleroderma, whereas recent studies describe US as a helpful objective marker in patients with psoriasis. The treatment effect in atopic patients observed using HFUS (20 MHz) was first described by [10]. The presence of a subepidermal low echogenic band (SLEB) in visually healthy skin enables skin lesion differentiation. Its thickness correlates with the histological degree of epidermal hyperkeratosis and intensity of inflammatory infiltrates [2,7,10,11]. Although different studies [2,11] examine the validity, repeatability, and reliability of skin measurements, which correlate with histological analysis, none of them analyze the HFUS capability in skin lesion differentiation. Such a possibility can be essential in the case of neoplastic or inflammatory skin diseases. The US-based assessment of the therapeutic decision is especially important in the latter, where the early differentiation between atopic dermatitis (AD) and psoriasis is crucial for the applied therapy. The classification step can also be significant for further image processing algorithms in image segmentation and image-based measurements.

Standalone visual analysis of HFUS images does not provide reliable results. The problem with HFUS skin image classification is its interpretation, usually followed and supported by visual or dermatoscopic analysis of the affected skin combined with medical history and interview. By analyzing the HFUS image only, the expert can rarely choose between AD, psoriasis, or healthy skin. On the other hand, the computer-aided diagnosis (CAD) system, which enables automated HFUS image classification, can support the physicians in their final decision. Moreover, further accurate image data analysis in different skin pathologies can strongly rely on the initial classification. Individual diseases require dedicated analysis, for example, the segmentation of particular structures, for example, tumors, the SLEB layer, epidermis layer, and so forth. The HFUS classification step should be considered an integral part of CAD in dermatology. Therefore, we decided to introduce a deep learning-based approach for this; the first in this field. Possible reasons for the absence of HFUS skin classification can be identified due to the relative novelty of HFUS in dermatology, a limited number of available datasets and experts able to analyze and annotate the data, or difficulties in the clear translation of the classical US interpretation into HFUS-based diagnosis.

Despite the lack of algorithms for HFUS image classification, different methods have appeared for automated and semi-automated HFUS (or UHFUS) image analysis. Pereyra et al. [12] first described the segmentation of skin lesions in a 25 MHz US using a generalized Rayleigh mixture model, then References [7,13,14,15,16] presented automatic skin layer segmentation algorithms. They employed classic segmentation approaches such as level sets and active contour models [14,15,16], or the most recent deep learning-based solutions [7]. The latter is now widely used in image segmentation, mainly through convolutional neural networks (CNNs), consisting of different layers responsible for dedicated data processing [17]. Skin layers were also segmented in optical coherence tomography (OCT) data [18,19,20,21,22], where the latest approach [22] involved the deep U-Net architecture, followed by the Savitzky–Golay filter and Fourier domain filtering.

Deep neural networks are also now common in the conventional US (<20 MHz) image analysis. Huang et al. [23] described an application for breast and liver lesion diagnosis, fetal and cardiovascular image classification, or as a thyroid nodule diagnosis support. Liu et al. [1] added kidney, bone, prostate, and brain US image processing to this list, with CNNs mainly used for classification tasks. Cai et al. [24] underlined the importance of deep learning in US image analysis of breast nodules. The most applicable architectures in US data classification are GoogLeNet, introduced by Han et al. [25] to classify breast lesions and by Chi et al. [26] for thyroid nodules, VGGNet and fully-connected networks (FCN) to differentiate the level of liver fibrosis [27], or Inception-v3, ResNet-101, and DenseNet-169, achieving the best performance in the automatic classification of common maternal–fetal ultrasound planes [28].

Due to the problem with the access to the training data, the models above cannot mostly generalize knowledge to the unknown clinical data (acquired in different medical fields, conditions, acquisition protocols, etc.). Hence, various ideas were introduced to handle generalization and overfitting issues, for example, semi-supervised learning, transfer learning, learning from noisy labels and learning from computer-generated labels [29]. Transfer learning (TL) seems to be the most common. Van Opbroek et al. [30] reported that TL improves supervised image segmentation across imaging protocols in medical applications. It employs a model pre-trained in a source domain and then fine-tunes it to the target task [29]. Morid et al. [31] provided the summary of methods employing TL to medical image analysis using the CNNs trained on the non-medical ImageNet dataset [32]. Ayana et al. [33] limited the review to US imaging of breast cancer.

The fast development of DL approaches enables the extraction of discriminative image features to classify different image modalities. The most common solution, CNN, automatically learns a representation of visual features from the input image textures [34] providing superior performance compared to other conventional machine learning methods. However, the integration of intelligent systems into clinical practice requires a visual explanation for their decision [35]. Therefore, Zhou et al. [36] proposed class activation mapping (CAM) to indicate discriminative image regions used by the CNN in image data classification. Selvaraju et al. [35] extended this work, proposing a Grad-CAM method applicable to a broader range of CNN models, including CNNs with fully connected layers. An application of Grad-CAM to medical images was given in [34], where the authors proposed class-selective relevance mapping (CRM) for localizing and visualizing discriminative regions of interest (ROIs) in the multimodal images.

Due to the absence of classification frameworks for HFUS skin image data, we present such a solution in our work. The HFUS images of the skin are similar in different pathologies, so it is difficult to define a set of features that would enable their classification. Since CNNs reach for an extensive collection of primitive features generalized in subsequent layers, they seem well suited to this problem. This is the first work addressing HFUS skin image classification. placeholder The proposed framework starts with the epidermis segmentation step to reduce the region of interest, followed by estimating a skin layer map. Then, the classification step is verified with a dedicated procedure using the Grad-CAM algorithm and the previously obtained skin layer map. Our novel approach combines segmentation and classification, providing the final results with the estimated classification confidence level. The method was verified with a database consisting of 631 HFUS images of AD (303), psoriasis (77), or neoplastic diseases (200), with a control group of 51 subjects, all described by an expert in a multi-stage diagnosis. The experiments included the study of the segmentation step, the influence of ROI reduction with different parameters, CNN architecture selection, and Grad-CAM-based model verification.

The datasets used for training and validation are specified in Section 2.1. The classification, epidermis segmentation, and classification confidence estimation are presented in detail in the remainder of Section 2. Section 3 describes the training and validation experiments along with the results. Due to the lack of state-of-the-art methods we can directly compete with, we assess our model in various architecture modes and flowchart parameter settings. The study is discussed in Section 4, and Section 5 concludes the paper.

## 2. Materials and Methods

### 2.1. Materials

To design a classification algorithm of different skin diseases, we combined three HFUS datasets. The first one, further called benchmark, consists of 380 HFUS images of 380 patients with atopic dermatitis (303 images) or psoriasis (77). The dataset is publicly available [37] along with the pre-trained SegUnet model for skin layer segmentation [7]. The second dataset consists of 200 images of 32 patients with different non-melanocytic skin tumors: BCC (143 images), fibroma (32), skin metastasis of breast cancer (10), keratofibroma (9), superficial BCC (3), and squamous cell carcinoma (3), whereas the third data set includes 51 images of 51 patients with healthy skin. All patients were Caucasian, men and women aged 18–86. The age in individual groups corresponds to the frequency of particular dermatological problems in specific age groups: mainly young adults in AD and psoriasis (18–45) and the elderly in skin tumors (69–86), with a broader range in the control group (26–65). The medical diagnosis for all patients was provided by an expert in dermatology, based on visual analysis of the skin, dermatoscopic image analysis, medical history, and medical interview. Thus, the methodology was designed, trained, and tested using a total of 631 HFUS images in four groups: AD, psoriasis, skin tumor, and control group.

**Figure 1:**
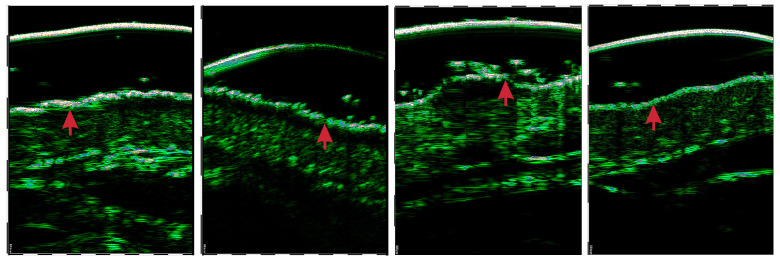
Exemplary HFUS images of four classes under consideration: (from left) AD, psoriasis, skin tumor, and control group.

All the analyzed HFUS image data were acquired using DUB SkinScanner75 with a 75 MHz transducer. The images have the same size of 2067×1555 pixels, but different resolutions (lateral × axial): 0.0019×0.085, 0.0024×0.085, 0.0031×0.085 mm/pix. The images were acquired at the affected skin area or forearm, in the case of the control group. The images were captured perpendicularly to the skin surface (±10 degrees) with minimal pressure from the physician (the DUB SkinScanner75 forces the user to position the probe to the right angle). The epidermal layer was delineated by one expert and verified by another in all 580 images of AD, psoriasis, and skin lesions. Exemplary images of four considered classes are shown in Figure 1.

### 2.2. HFUS Skin Image Classification

**Figure 2:**
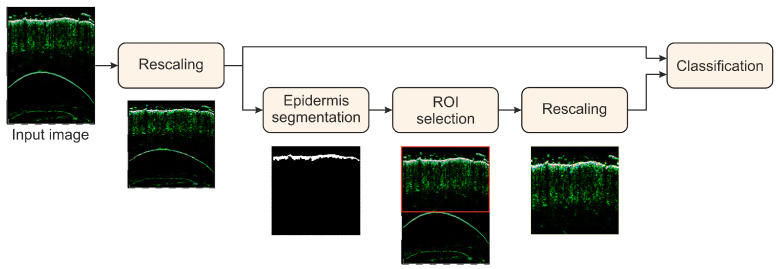
General scheme of the skin classification method.

The overall goal of the study was to develop and evaluate the HFUS image classification framework, which provides the most reliable classification results. The general scheme of the classification algorithm with two processing paths is shown in Figure 2. The first (top) path is based on the pre-trained and fine-tuned CNN model, leading directly to the final classification result. During the experiments, we considered five CNN models: DenseNet-201 [38], GoogLeNet [39], Inception-ResNet-v2 [20], MobileNet [40], and ResNet-101 [41], all pre-trained on the ImageNet dataset, and fine-tuned with our HFUS images of skin diseases. Moreover, two different augmentation setups were introduced to secure classification robustness.

In the second (bottom) path, we extract the ROI from the image to feed the classifier with the skin-layers-related content. For this, we first introduce the epidermis segmentation to our framework, as the epidermis layer is rather clearly visible in each HFUS image (see Figure 1). The epidermis segmentation is performed with the DeepLab v3+ network built on a pre-trained backbone for feature extraction [42,43]. Different models (trained via transfer learning) were tested to serve as a backbone: ResNet-18, ResNet-50 [41], and Xception [44]. Originally, the models were pre-trained using the ImageNet database [32,45]. Moreover, the model described in [7] trained for the benchmark database was considered as the possible choice at this step. Finally, we selected the DeepLab v3+ model with an Xception as the backbone, pretrained on ImageNet database, with the stochastic gradient descent with momentum optimizer (SGDM) as the most efficient in a series of experiments. Quantitative inter-model comparison can be found in Section 3. We used data augmentation through random geometric transformations (horizontal reflection, ±10-pixel translation in both directions). The batch size for training was set to 8, and the maximum number of epochs to 200. The loss function we employed was weighted generalized Dice loss [46], able to handle possible *epidermis*/*background* pixel distribution imbalance.

Based on the epidermis segmentation mask, the ROI for further analysis is selected. This procedure is an approach to unify the images before the main classification procedure. It addresses the gel and probe membrane appearance and standardizes the positioning of skin layers in the image. Depending on the experiment, we reduce the image area above and below the epidermis. The considered variants of ROI include: (1) removing the upper part of the image 30 pixels above the top pixel of the segmented epidermis layer; (2) removing the upper part of the image 1 mm above the top pixel of the epidermis; (3,4) removing the upper part as in (1) and (2), respectively, and removing the lower part 2 mm below the bottom pixel of the epidermis. Additionally, we set to 0 all pixels in all the areas in the ROI, denoted as avoided in the skin layer map (see Section 2.3.2).

### 2.3. Classification Confidence Estimation

**Figure 3:**
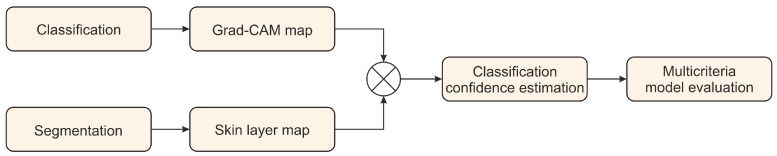
General scheme of the classification model confidence estimation method.

In our study, we designed a methodology to assess the reliability of a classification model (Figure 3). It relies on two components: the Grad-CAM deep learning interpretability technique developed by Selvaraju et al. [35] and a skin layer map estimate based on our epidermis segmentation (Figure 2). The method produces a classification confidence level (CCL) measure to assess individual classification results quantitatively. Moreover, we introduce a dedicated multicriteria model evaluation procedure to evaluate the model robustness and reliability.

#### 2.3.1. Grad-CAM Map

The Grad-CAM deep learning interpretability technique was proposed by Selvaraju et al. [35] to provide a visual explanation of the deep model decision. It employs the gradient of the classification score with respect to the final feature map. Grad-CAM enables the identification of parts of the input image with the greatest impact on the classification results. According to [35], the Grad-CAM map can be estimated as:

(1) $$L^{c}=ReLU\left(\sum_{k}\alpha_{k}^{c}A^{k}\right),$$

where *c* is a target class (we use the class resulting from the CNN-based classification), $\alpha_{k}^{c}$ are neuron ’importance’ weights of a feature map *k* for a target class *c*, calculated as:

(2) $$\alpha_{k}^{c}=\frac{1}{Z}\sum_{i}\sum_{j}\frac{\partial y^{c}}{\partial A_{ij}^{k}},$$

where $A^{k}$ stands for the feature map of the final convolutional layer, *Z* comes from its size, and $\frac{\partial y^{c}}{\partial A^{k}}$ is the gradient of the score for class *c*, $y^{c}$ (before the softmax layer), with respect to the feature map $A^{k}$ of the final convolutional layer.

#### 2.3.2. Skin Layer Map

In their original work, Selvaraju et al. [35] provide a meaningful example of the classified objects (cat and dog), which, combined with a Grad-CAM map, enables visual evaluation of the network focus in the image. The problem with HFUS image classification is its visual interpretation. It is challenging to clearly define the ROI, which should be the network interest in target skin diseases. However, based on semantic interpretation and human understanding of HFUS images, we propose a skin layer map (SLM) to indicate regions, which are considered during medical diagnosis, and other regions that the network should not focus on to avoid overfitting.

**Figure 4:**
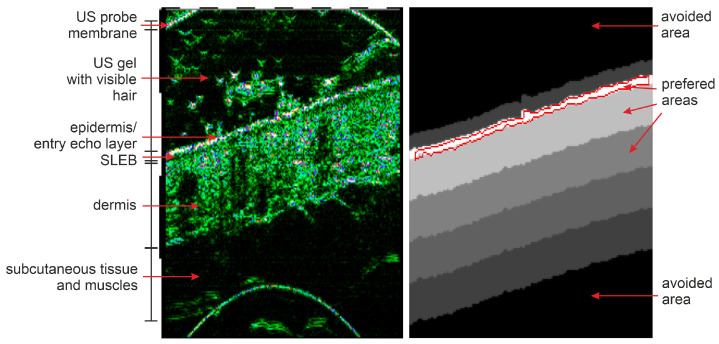
Illustration of a skin layer map (**right**) obtained for the HFUS image (**left**) based on the epidermis segmentation results (delineated in red).

Based on the epidermis segmentation outcome and skin layer thicknesses reported in [47,48] and measured in our database, we create the individual skin layer map for each input image. The map indicates the most relevant regions that should be considered in skin diagnosis and gain particular focus from the CNN. As the epidermis area results directly from the segmentation step, the dermis region location is estimated based on the thickness values given in [47,48] and measured in our dataset. The reported skin (epidermis and dermis) thickness in different interesting body parts varies from 1.5 mm to 2.5 mm [47]. Similar values can be observed in our dataset, where this thickness falls below <2 mm. On the other hand, the regions of the epidermis, possible SLEB, and upper dermis area are essential for our findings. Considering this and assuming that the skin layers are mostly parallel (with some exception in the skin tumor area), we generate the SLM as illustrated in Figure 4. Starting from the epidermis segmentation results, where the SLM has its maximum of 3, it decreases twice by 1 every 0.5 mm (half of the dermis layer thickness), and then twice by 0.5 every 0.5 mm until the dermis ends. Moreover, it is set to 0 in the US gel area located directly above (30 pixels) the epidermis and −1 in the image regions, which should be avoided in further analysis. The avoided parts include the US probe membrane, remaining US gel area, or muscles.

#### 2.3.3. Classification Confidence Level

**Figure 5:**
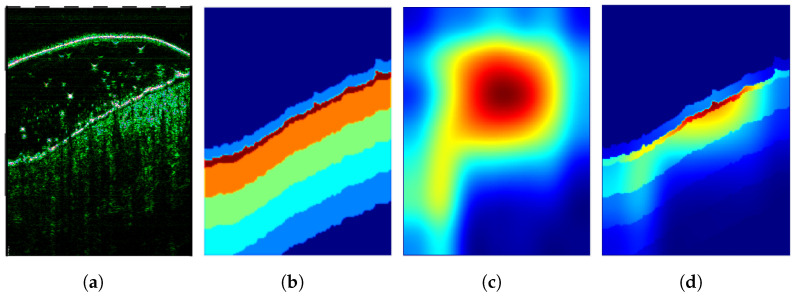
Illustration of the classification confidence map (**d**) based on the input image (**a**), skin layer map (**b**), and Grad-CAM map (**c**).

To assess the reliability of classification, we combine the Grad-CAM outcome and our SLM to get the classification confidence map and classification confidence level $t_{\kappa }$ for a classification model κ (see Figure 5). Let $L_{\kappa }\left(i,j\right)$ and $H_{\kappa }\left(i,j\right)$ be the Grad-CAM map value and the SLM value, respectively, corresponding to the input image pixel I(i,j). Assuming that $L_{\kappa }$ is normalized for each image to the range of 0 to 1, the CCL is given as:

(3) $$t_{\kappa }=\frac{1}{M}\sum_{k,l}^{M}G_{\kappa }\left(k,l\right),$$

where:

(4) $$G_{\kappa }=\left\{L_{\kappa }\left(i,j\right)H_{\kappa }\left(i,j\right):\;L_{\kappa }\left(i,j\right)H_{\kappa }\left(i,j\right)>0,\;i\in \left\{1,2,\ldots ,m\right\},\;j\in \left\{1,2,\ldots ,n\right\}\right\},$$

m×n is the input image size, and *M* stands for the cardinality of $G_{\kappa }$. Note that due to different sizes of the original matrices, the obtained Grad-CAM maps are rescaled to the original image size and eventually cropped to match the ROI before applying (3) and (4).

#### 2.3.4. Multicriteria Model Evaluation

**Figure 6:**
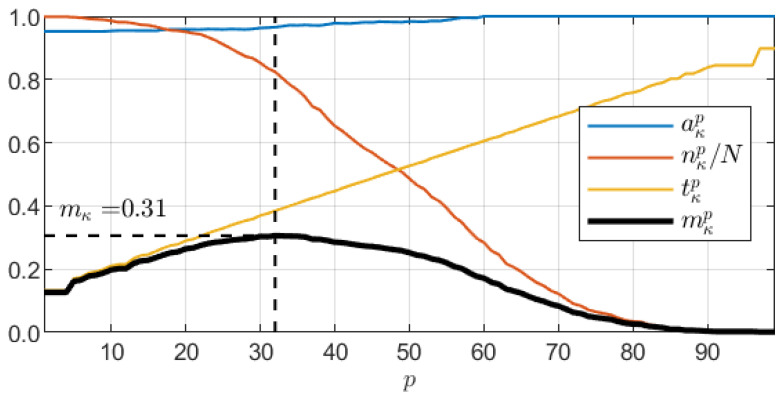
Illustration of a multicriteria model evaluation measure $m_{\kappa }$ with charts of $m_{\kappa }^{p}$ and its components as a function of cutoff percentile number *p*.

For the evaluation of a classification model κ, we propose a dedicated metrics $m_{\kappa }$ based on three components: the CCL value $t_{\kappa }$, classification accuracy $a_{\kappa }$, and test dataset size $n_{\kappa }$. Each of them should be as large as possible to secure high classification accuracy and reliability. The concept constituting multicriteria model evaluation aims at achieving high accuracy with high confidence over the largest possible dataset. $m_{\kappa }$ is determined through maximization:

(5) $$m_{\kappa }=\max_{p}\left(m_{\kappa }^{p}\right),$$

(6) $$m_{\kappa }^{p}=a_{\kappa }^{p}\frac{n_{\kappa }^{p}}{N}t_{\kappa }^{p},$$

where N=631 is the number of images in the entire dataset. p=1,2,…,99 is used to determine the CCL threshold $t_{\kappa }^{p}$ as the *p*th percentile of CCL values produced by the model *c* over the entire dataset. In each iteration, we limit the dataset to $n_{\kappa }^{p}$ samples with the CCL value greater than $t_{\kappa }^{p}$ and calculate partial accuracy $a_{\kappa }^{p}$. Thus, with increasing *p*, the dataset size decreases, while $t_{\kappa }^{p}$ increases. However, since such a procedure in general preserves strong samples, the classification accuracy over the limited dataset is also non-decreasing. Figure 6 shows sample charts of all four measures over a certain model κ.

## 3. Experiments and Results

The training and experiments were performed in the Deep Learning Toolbox (version 14.2) of the Matlab software (9.10.0.1602886, R2021a) on a workstation with an 8-Core CPU @ 3.20 GHz, 64 GB RAM, and Nvidia Quadro RTX 6000 24 GB GPU.

### 3.1. Epidermis Segmentation

As introduced in Section 2.3.2, the skin layer map is determined based on the initial epidermis segmentation. Thus, we verified different pre-trained (using ImageNet) models as a backbone for the DeepLab v3+ architecture: ResNet-18, ResNet-50, and Xception. We trained each using two different loss functions: cross-entropy and Dice loss. The data augmentation involved random geometric transformations (horizontal reflection, ±10-pixel translation in both directions). We also compared the obtained results with the SegUNet model described in [7]. To assess the segmentation, we employed the Dice index [49], commonly used to measure the spatial overlap in medical imaging studies, including HFUS analysis [7,15,50]. Since the epidermal layer was delineated by the expert in 580 images of AD, psoriasis, and skin tumors, the experiments were limited to this subset.

**Figure 7:**
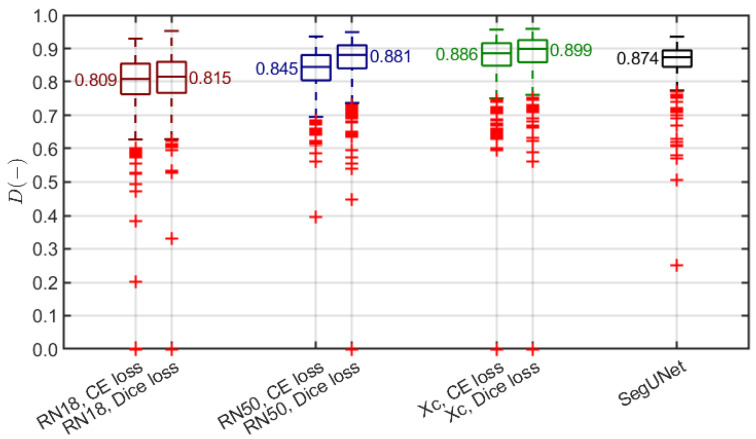
Summary of epidermis segmentation results (Dice index). Red, blue and green plots present our method with different pre-trained models used as a backbone of the DeepLab v3+ architecture (ResNet-18, ResNet-50, Xception) trained using cross-entropy loss and Dice loss. Black plot refers to the SegUNet model with the top performance in [7]. Each box covers 25th to 75th percentile confidence interval with median indicated by a central line. Whiskers refer to 1.5 times the interquartile range. Outliers are indicated with red +.

We used the external 10-fold cross-validation to assess the segmentation. The non-testing remaining data were divided into training and validation subsets (8:1 ratio) in each experiment. Moreover, we ensured that the image data of a single patient from the neoplastic dataset (200 images of 32 patients) were used only for either training or testing, never being shared by both subsets. The results are shown in Figure 7. Two observations are evident. The DeepLab v3+ model with the backbones pre-trained on ImageNet outperforms the SegUNet from [7], and the Dice-loss-based training is more efficient compared to the cross-entropy loss. Hence, we selected the DeepLab v3+ model with an Xception backbone, SGDM optimizer, and Dice loss for the epidermis segmentation.

### 3.2. Classification

For skin classification, we considered five different CNN models, offering the most promising effectiveness in numerical experiments: DenseNet-201 [38], GoogLeNet [39], Inception-ResNet-v2 [20], MobileNet [40], and ResNet-101 [41], all pre-trained on the ImageNet database. Two modes of augmentation were used. In the first mode (denoted as Aug0), we applied random geometric transformations: horizontal reflection, ±10-pixel translation in both directions, similar to the one used in training the epidermis segmentation model. The second mode (Aug1) additionally employed random $\pm 20^{\circ }$ rotation. Again, we used the external 10-fold cross-validation scheme with the non-testing data divided into training and validation subsets (8:1) in a patient-wise mode. The batch size for training was set to 8, and the maximum number of epochs to 50 with the SGDM optimizer and a cross-entropy loss. Additionally, we analyzed the ROI selection influence on the classification results. We considered all the four options for ROI extraction described in Section 2.2 with the variant numbers (1)–(4) given there used in all tables in this section. The results obtained by models fed with the original image are denoted as (0).

**Figure 8:**
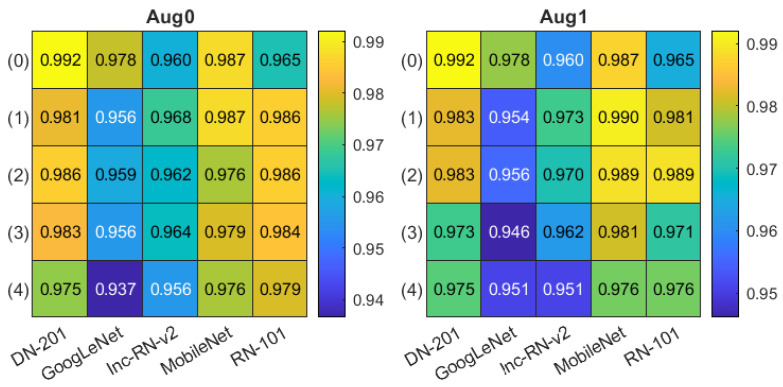
Classification accuracy summary in two data augmentation options Aug0, Aug1. Columns correspond to different deep models, rows refer to ROI extraction modes (described in the text).

**Figure 9:**
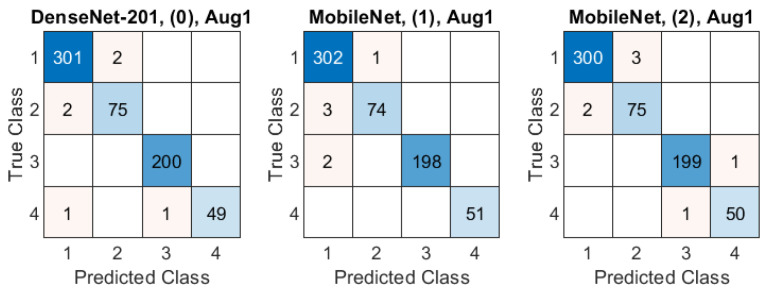
Confusion matrices in three best-case classification models from Figure 8. Class IDs: 1–AD, 2–psoriasis, 3–skin tumor, 4–control group. Models are specified above each matrix in a format: model name, ROI extraction mode, data augmentation mode.

The classification accuracy obtained in all experiments are presented in Figure 8. The Aug0 and Aug1 in Figure 8 refer to data augmentation modes. Tables’ rows refer to the ROI extraction modes (0)–(4) described in Section 2.2, whereas columns correspond to different deep models. The highest accuracies (0.992) obtained in both data augmentation modes by DenseNet-201 over the original-size images are highlighted in yellow. The confusion matrices for three best models (in terms of the highest accuracy) from Figure 8 are presented Figure 9: DenseNet-201, no ROI extraction (0), Aug0; MobileNet, ROI mode (1), Aug1, and MobileNet, ROI mode (2), Aug1.

### 3.3. Classification Confidence Estimation

**Figure 10:**
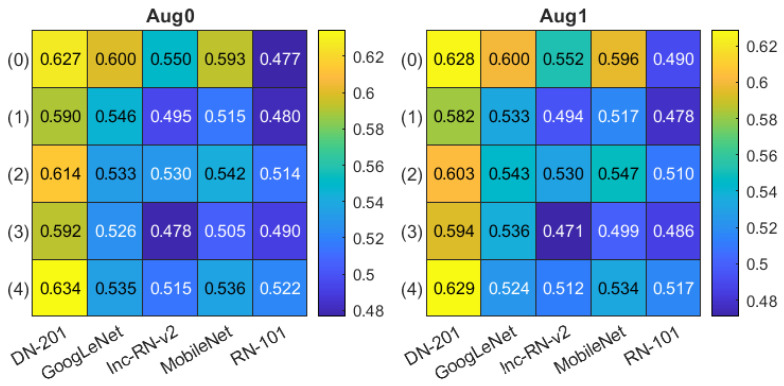
Mean classification confidence level $t_{\kappa }$ summary in two data augmentation options Aug0, Aug1. Columns correspond to different deep models, rows refer to ROI extraction modes (described in the text).

The mean classification confidence levels $t_{\kappa }$ for each model investigated in Section 3.2 are given in Figure 10 (organized in correspondence with Figure 8). The highest $t_{\kappa }$ value of 0.634 was obtained for DenseNet-201, for ROI in mode (4) with Aug0. The accuracy of that particular model was 0.975 (Figure 8), lower than the top accuracy (0.992).

### 3.4. Multicriteria Model Evaluation

**Figure 11:**
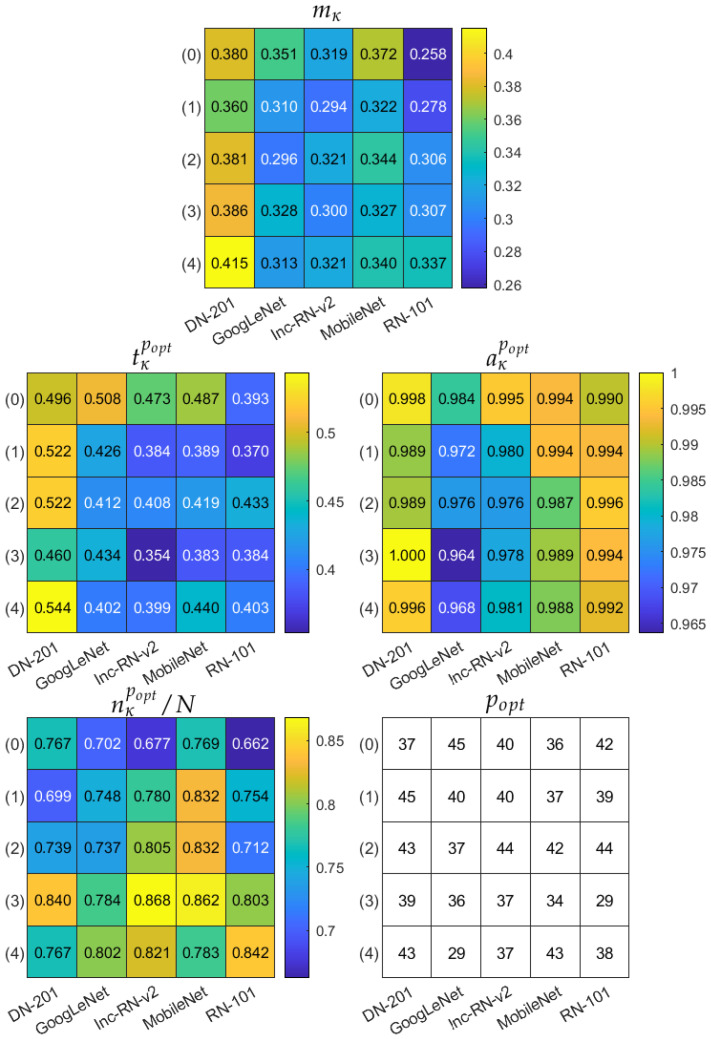
Multicriteria model evaluation summary in data augmentation option Aug0. Top table: $m_{\kappa }$ measure. Remaining tables present components of $m_{\kappa }$ obtained for $p_{opt}=\arg\max_{p}\left(m_{\kappa }^{p}\right)$ and $p_{opt}$ itself. In each table, columns correspond to different deep models, rows refer to ROI extraction modes (described in the text).

**Figure 12:**
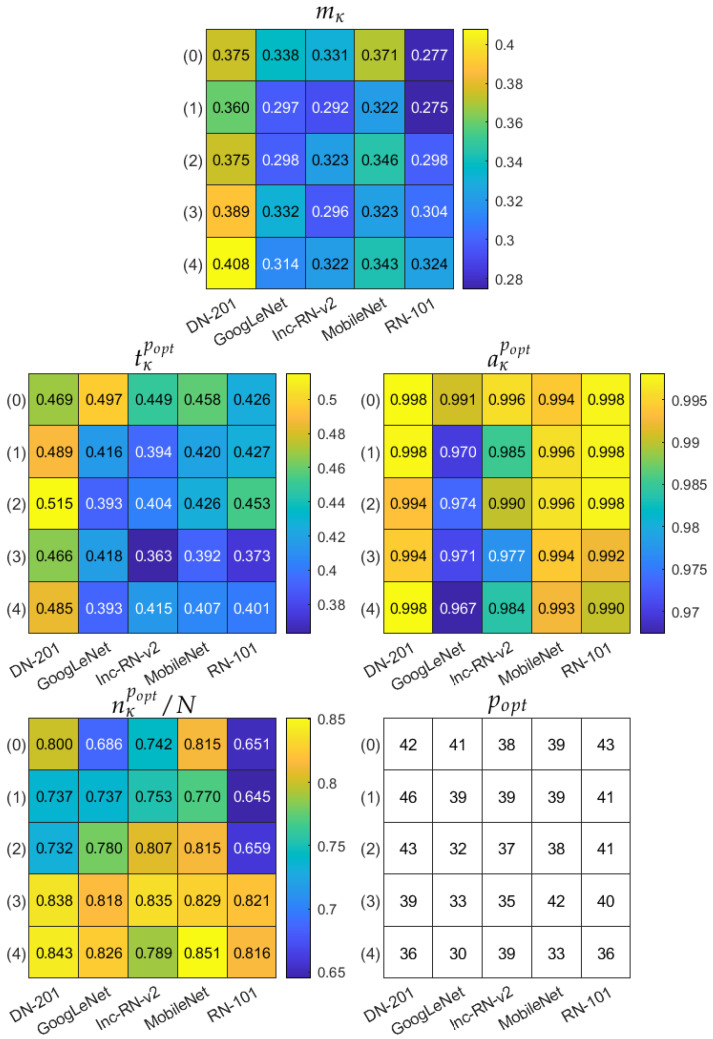
Multicriteria model evaluation summary in data augmentation option Aug1. Top table: $m_{\kappa }$ measure. Remaining tables present components of $m_{\kappa }$ obtained for $p_{opt}=\arg\max_{p}\left(m_{\kappa }^{p}\right)$ and $p_{opt}$ itself. In each table, columns correspond to different deep models, rows refer to ROI extraction modes (described in the text).

**Figure 13:**
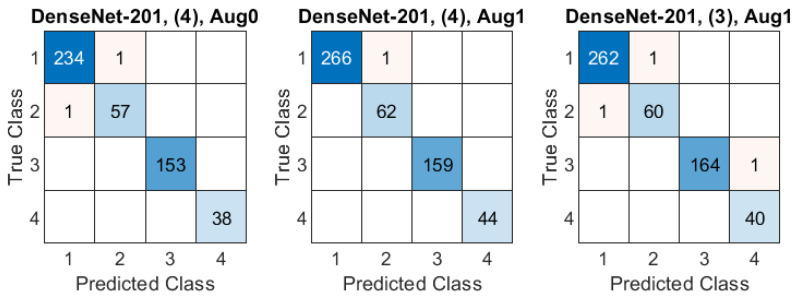
Confusion matrices in three best-case classification models from Figure 11 and Figure 12 over a limited dataset with respect to the $m_{\kappa }$ value. Class IDs: 1–AD, 2–psoriasis, 3–skin tumor, 4–control group. Models are specified above each matrix in a format: model name, ROI extraction mode, data augmentation mode.

Finally, we apply the multicriteria model evaluation measure $m_{\kappa }$ to the trained models. The results are gathered in Figure 11 and Figure 12, corresponding to two augmentation modes Aug0 and Aug1, respectively. In both figures, the top table refers to the $m_{\kappa }$ measure, and the remaining tables present the corresponding partial accuracy $a_{\kappa }^{p_{opt}}$, data subset size $n_{\kappa }^{p_{opt}}/N$, and classification confidence level $t_{\kappa }^{p_{opt}}$, all obtained for $p_{opt}=\arg\max_{p}\left(m_{\kappa }^{p}\right)$. The bottom right table shows the $p_{opt}$ itself. The multicriteria model evaluation indicates DenseNet-201, with ROI in mode (4) and Aug0 data augmentation as the most robust model, reaching 0.996 accuracy over 76.7% of data, for which the CCL is not less than 0.544. Confusion matrices for three best models in terms of $m_{\kappa }$ are shown in Figure 13 over the subset limited by $p_{opt}$: DenseNet-201, ROI mode (4), Aug0; DenseNet-201, ROI mode (4), Aug1, and DenseNet-201, ROI mode (3), Aug1.

## 4. Discussion

The methodology presented in this study mainly focuses on the classification of normative and pathological skin in HFUS images; yet, in the first step, we developed a method for the robust segmentation of the epidermal layer. The results were used for two purposes: to extract the meaningful ROI for classification and to enable reliable validation of classification confidence.

We prepared a deep model through transfer learning for segmentation, mainly due to the limited dataset size. With various deep semantic segmentation architectures under consideration, the experiments favored the DeepLab v3+ model with a pre-trained Xception backbone. The selection is justified by the results of Dice-index-based validation given in Figure 7. Since the Dice index is a reference measure in medical image segmentation, we were able to compare our results to rare previous research in this field. The transfer learning approach turned out to be more efficient than the best method presented in [7]. Thus, we find the transfer learning promising for possible future research on the segmentation of either epidermis or other skin layers.

**Figure 14:**
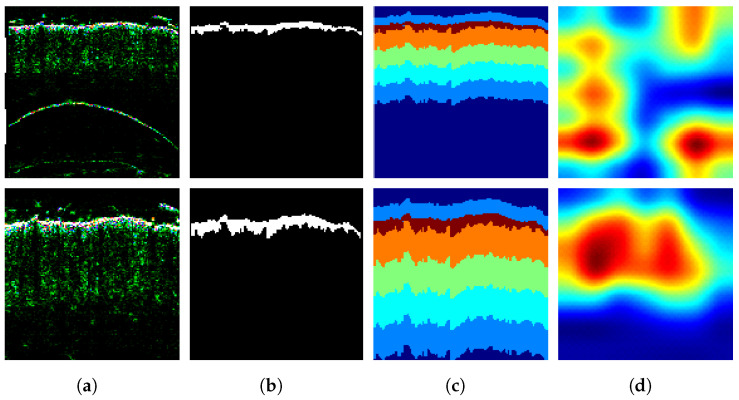
Illustration of Grad-CAM maps (**d**) obtained for the original image (top) and the extracted ROI (bottom) (**a**), with the corresponding epidermis segmentation results (**b**), and skin layer maps (**c**).

Despite a generally good accuracy (over 0.93), the classification assessment over the entire dataset indicated some differences between models and ROI extraction or data augmentation modes (Figure 8), consistently favoring the DenseNet-201 ahead of the MobileNet. Most frequent misclassifications were related to AD and psoriasis cases—see examples in Figure 9. To ensure that the models focus on the meaningful regions of the image, we took a closer look at the classification process using the Grad-CAM tool. As a result, we could notice multiple cases where the networks focus on the gel layer or other parts instead of the skin. An example can be seen in Figure 14, where the same image was analyzed without (top) and with the ROI extraction (bottom). The Grad-CAM heatmap in the latter seems to cover the skin layers more adequately.

**Figure 15:**
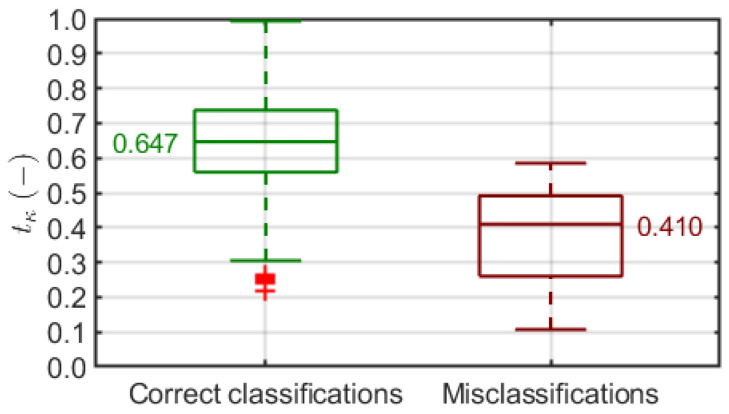
Distribution of classification confidence level $t_{\kappa }$ in correct and incorrect classification groups. Each box covers 25th to 75th percentile confidence interval with median indicated by a central line. Whiskers refer to 1.5 times the interquartile range. Outliers are indicated with red +.

To address this matter, we introduced a classification confidence level measure to reward classifications where the Grad-CAM heatmap matches the skin region estimated layer-wise based on the epidermis segmentation outcome. The mean CCL distribution over all models and setups (Figure 10) did not necessarily correspond to the classification accuracy distribution (Figure 8). On the other hand, we noticed a significant correlation between the CCL value and classifier decision in individual cases. Figure 15 shows the distributions of $t_{\kappa }$ in correct and incorrect classification groups produced by one of the models. Low $t_{\kappa }$ is clearly more likely to lead to a wrong classification, whereas a high enough $t_{\kappa }$ guarantees the right decision. We observed such a relationship consistently in different models, and we find it a reliable justification for the use of Grad-CAM-based classification confidence assessment.

Moreover, we extended our model evaluation methodology by introducing in Section 2.3.4 an additional multicriteria measure $m_{\kappa }$ that follows the increasing CCLs produced by the model over the test dataset. As we know from Figure 15, dropping off low-CCL cases should result in increasing accuracy over a limited dataset since the misclassifications should be excluded early. Our measure indicates the model that maximizes Equation (6), which balances classification accuracy, confidence, and dataset size. The multicriteria evaluation points out the DenseNet-201 model with the Aug0 data augmentation, fed by the extracted ROI in the (4) option (1 mm from the top, 2 mm dermis) with the top $m_{\kappa }$ value of 0.415. In this case, the classification accuracy $a_{\kappa }^{p_{opt}}$ reaches 0.996 with $a_{\kappa }^{p_{opt}}=0.544$ over 76.7% of the dataset (Figure 11). Note that this model’s accuracy over the entire dataset was 0.975 with a mean CCL of 0.634 (Figure 8 and Figure 10).

The multicriteria model evaluation strategy attempts to handle outlier cases without manually pointing them out or removing them before the analysis. The latter would require defining additional image quality constraints, and so forth. Moreover, there is not much room to reduce datasets in deep learning: the smaller the dataset, the more questionable robustness of the model, which contradicts the original goal. In our approach, the number and character of outlier cases are automatically reflected after being processed by the model, enabling its quantitative assessment. We believe that such an idea can be widely used to design more reliable deep models not only for CAD applications.

Our results prove that the map of skin layers based on the initial epidermis segmentation combined with Grad-CAM can be used to evaluate the skin classification in HFUS. However, the individual layers’ thickness may be affected by other factors, for example, the measurement location on the patient’s body, gender, or body mass index. Hence, a personalized skin map based on a medical interview or automated segmentation of all skin layers (which is rather challenging for now) is likely to improve the analysis.

## 5. Conclusions

This paper presents a novel CNN-based framework for the HFUS image classification, with a multicriteria model evaluation. It enables both classifying the HFUS data into four groups and evaluating the obtained results with a Grad-CAM-based tool. It is the first method targeting HFUS image classification combining inflammatory skin diseases and skin tumors and the first CNN application for this task. The obtained results prove that such an analysis is accurate and reliable, whereas the additional introduced procedures: epidermis segmentation, followed by skin layer map estimation and multicriteria model evaluation, enable the reliable validation of the method.

## Data Availability

Data sharing is not applicable to this article.
